# Publisher Correction: A TonB-dependent receptor constitutes the outer membrane transport system for a lignin-derived aromatic compound

**DOI:** 10.1038/s42003-019-0724-8

**Published:** 2019-12-17

**Authors:** Masaya Fujita, Kosuke Mori, Hirofumi Hara, Shojiro Hishiyama, Naofumi Kamimura, Eiji Masai

**Affiliations:** 1 Department of Bioengineering, Nagaoka University of Technology, Nagaoka, Niigata Japan; 2 Department of Chemical Process Engineering, Malaysia-Japan International Institute of Technology, Universiti Teknologi Malaysia, Kuala Lumpur, Malaysia; 3 Forestry and Forest Products Research Institute, Tsukuba, Ibaraki Japan

**Keywords:** Applied microbiology, Bacterial genetics

Correction to: *Communications Biology* 10.1038/s42003-019-0676-z, published online 22 November 2019.

In the original published version of the article, a correction to Figure 2c was not carried out prior to publication. All samples were incorrectly labelled as having been treated with 1 mM DDVA. The leftmost lane for SYK-6, *∆ddvT* and *∆ddvR* samples shown in the blot should have been marked as untreated. The error has been corrected in the HTML and PDF versions of the article.

