# Supplementary material for: A TonB-dependent receptor constitutes the outer membrane transport system for a lignin-derived aromatic compound
Source: Commun Biol. 2019 Nov 22;2:432. doi: 10.1038/s42003-019-0676-z (PMC6874591; doi:10.1038/s42003-019-0676-z)
Supplement: Supplementary file 1 — Supplementary information [file 42003_2019_676_MOESM1_ESM.pdf]

Supplementary Table 1. Known TBDR genes used for the phylogenetic analysis

Abbreviation	Gene	Accession No.	Strain	Reference
*St*_BtuB	*btuB*	AAL22968	*Salmonella typhimurium* LT2	1
*Ec*_BtuB	*btuB*	AAA23524	*Escherichia coli* K-12	2
*Cc*_BtuB	*btuB*	ACL95291	*Caulobacter crescentus* NA1000	3
*Bp*_FauA	*fauA*	Q9X6A5	*Bordetella pertussis*	4
*Ye*_FcuA	*fcuA*	Q05202	*Yersinia enterocolitica*	5
*Ec*_FepA	*fepA*	BAA35225	*Escherichia coli* K-12	6
*Ec*_FhuA	*fhuA*	AAC73261	*Escherichia coli* K-12	7
*Ec*_FhuE	*fhuE*	AOR19445	*Escherichia coli* K-12	8
*Ye*_FoxA	*foxA*	CAA42975	*Yersinia enterocolitica* ssp. serotype 0:8, WA-C	9
*Pa*_FptA	*fptA*	AAG07609	*Pseudomonas aeruginosa* PAO1	10
*Pa*_FpvA	*fpvA*	AAG05786	*Pseudomonas aeruginosa* PAO1	11
*Ng*_FrpB	*frpB*	Q50944	*Neisseria gonorrhoeae*	12
*Hp*_FrpB4	*frpB4*	AAD08553	*Helicobacter pylori* 26695	13
*Pa*_FusA	*fusA*	CAG73790	*Pectobacterium atrosepticum* SCRI1043	14
*Sm*_HasR	*hasR*	Q79AD2	*Serratia marcescens*	15
*Hd*_HgbA	*hgbA*	AAP96732	*Haemophilus ducreyi* 35000HP	16
*Cc*_HutA	*hutA*	ACL95742	*Caulobacter crescentus* NA1000	17
*Vp*_IutA	*iutA*	BAB83802	*Vibrio parahaemolyticus* WP1	18
*Cc*_MalA	*malA*	ACL95835	*Caulobacter crescentus* NA1000	19
*Cc*_NagA	*nagA*	ACL93922	*Caulobacter crescentus* NA1000	20
*P*s_PbuA	*pbuA*	CAA51812	*Pseudomonas* sp. strain M114	21
*Pa*_PfeA	*pfeA*	AAG06076	*Pseudomonas aeruginosa* PAO1	22
*Pp*_PupA	*pupA*	CAA39942	*Pseudomonas putida* WCS358	23
*Pp*_PupB	*pupB*	CAA51995	*Pseudomonas putida* WCS358	24
*Sm*_RhtA	*rhtA*	AGG70967	*Sinorhizobium meliloti* 2011	25
*Sd*_ShuA	*shuA*	AAC27809	*Shigella dysenteriae* O-4576	26
*Cc*_SucA	*sucA*	ACL94659	*Caulobacter crescentus* NA1000	27
*Xa*_SuxA	*suxA*	AAM42628	*Xanthomonas campestris pv. campestris* str. ATCC 33913	28
*Ng*_TbpA	*tbpA*	AAA25503	*Neisseria gonorrhoeae* FA19	29
*Hd*_TdhA	*tdhA*	AAP95357	*Haemophilus ducreyi* 35000HP	30
*Nm*_ZnuD	*znuD*	AAF62323	*Neisseria meningitidis* MC58	31

Supplementary Table 2. Seventeen SYK-6 TBDR genes specifically induced during growth with lignin-derived aromatic compounds

Locus tag	Accession number	Substrate	Induction rate	*P*-value
SLG_02250	BAK64900.1	Acetovanillone	3.73	0.024
SLG_02360	BAK64911.1	Acetovanillone	3.34	0.020
SLG_04460	BAK65121.1	Protocatechuate	2.88	0.004
		DDVA	2.64	0.022
		GGE	2.20	0.071
		Syringate	3.51	0.049
SLG_07650	BAK65440.1	DDVA	4.87	0.009
SLG_09260	BAK65601.1	HMPPD	23.4	0.008
		DCA	3.41	0.007
SLG_09330	BAK65608.1	DCA	6.92	0.002
SLG_12400	BAK65915.1	Acetovanillone	11.9	0.009
SLG_16980	BAK66373.1	Acetovanillone	3.03	0.014
		Syringate	3.04	0.002
SLG_17580	BAK66433.1	Ferulate	3.50	0.004
SLG_24600	BAK67135.1	Acetovanillone	3.23	0.009
SLG_26390	BAK67314.1	HMPPD	5.80	0.001
SLG_27890	BAK67464.1	Pinoresinol	10.9	0.003
		HMPPD	3.14	0.020
		Acetovanillone	2.02	0.029
SLG_28190	BAK67494.1	Acetovanillone	2.19	0.005
SLG_31810	BAK67856.1	Acetovanillone	2.04	0.017
SLG_34070	BAK68082.1	Acetovanillone	2.27	0.004
		Ferulate	2.18	0.012
		Protocatechuate	2.17	0.001
SLG_36280	BAK68303.1	Protocatechuate	2.30	0.028
		Syringate	5.97	0.004
SLG_38050	BAK68480.1	DCA	35.4	0.001
		DDVA	2.00	0.013

GGE, guaiacylglycerol-β-guaiacyl ether; HMPPD, 1,2-bis(4-hydroxy-3-methoxyphenyl)-propane-1,3-diol; DCA, dehydrodiconiferyl alcohol; DDVA, 5,5′-dehydrodivanillate.

Supplementary Table 3. Strains and plasmids used in this study

Strains or plasmids	Relevant characteristic(s)[a]	Reference or source
Strains		
Sphingobium sp.		
SYK-6	Wild type; Nal^r Sm^r	32
SME002-3	SYK-6 derivative; Δ*ligI*; Nal^r Sm^r	33
SME048	SYK-6 derivative; Δ*ddvR*; Nal^r Sm^r	34
SME057	SYK-6 derivative; ΔSLG_38320 (*ompW*); Nal^r Sm^r	This study
SME096	SYK-6 derivative; ΔSLG_36940 (*tonB3*); Nal^r Sm^r	This study
SME097	SYK-6 derivative; ΔSLG_34540 (*tonB2*); Nal^r Sm^r	This study
SME099	SYK-6 derivative; ΔSLG_07650 (*ddvT*); Nal^r Sm^r	This study
SME100	SYK-6 derivative; ΔSLG_04460; Nal^r Sm^r	This study
SME109	SYK-6 derivative; ΔSLG_38050; Nal^r Sm^r	This study
SME133	SYK-6 derivative; Δ*ddvK*; Nal^r Sm^r	34
SME290	SYK-6 derivative; ΔSLG_37490 (*tonB4*); Nal^r Sm^r	This study
SME292	SYK-6 derivative; ΔSLG_01650 (*tonB5*); Nal^r Sm^r	This study
SME293	SYK-6 derivative; ΔSLG_14690 (*tonB6*); Nal^r Sm^r	This study
SME303	SYK-6 derivative; Δ*tonB3456*; Nal^r Sm^r	This study
SME304	SYK-6 derivative; Δ*tonB23456*; Nal^r Sm^r	This study
Escherichia coli		
HB101	*recA13 supE44 hsd20 ara-14 proA2 lacY1 galK2 rpsL20 xyl-5 mtl-1*	35
NEB 10-beta	*araD*139 Δ(*ara-leu*)7697 *fhuA lacX74 galK* (ϕ80 Δ*lacZ* Δ*M15*) *recA1 endA1 nupG rpsL* (Sm^r) Δ(*mrr-hsdRMS-mcrBC*)	New England Biolabs
Plasmids		
pRK2013	Tra$^+$ Mob$^+$ ColE1 replicon; Km^r	36
pJB861	RK2 ori broad-host-range expression vector; Km^r P_m *xylS*	37
pJB866	RK2 ori broad-host-range expression vector; Tet^r P_m *xylS*	37
pAK405	Plasmid for allelic exchange and markerless gene deletions in Sphingomonads; Km^r	38
pSEVA225	RK2 ori *lacZ* promoter probe broad host range vector; Km^r	39
pSEVA338	pBBR1 ori broad-host-range expression vector; Cm^r P_m *xylS*	39
pAK01650	pAK405 with a 1.9-kb deletion cassette carrying up- and downstream regions of *tonB5*	This study
pAK04460	pAK405 with a 2.0-kb deletion cassette carrying up- and downstream regions of SLG_04460	This study
pAK07650	pAK405 with a 2.0-kb deletion cassette carrying up- and downstream regions of *ddvT*	This study
pAK14690	pAK405 with a 2.1-kb deletion cassette carrying up- and downstream regions of *tonB6*	This study
pAK34550	pAK405 with a 2.0-kb deletion cassette carrying up- and downstream regions of *tonB2*	This study
pAK36940	pAK405 with a 1.8-kb deletion cassette carrying up- and downstream regions of *tonB3*	This study
pAK37490	pAK405 with a 2.3-kb deletion cassette carrying up- and downstream regions of *tonB4*	This study
pAK38050	pAK405 with a 2.0-kb deletion cassette carrying up- and downstream regions of SLG_38050	This study
pAK38320	pAK405 with a 2.0-kb deletion cassette carrying up- and downstream regions of *ompW*	This study
pJB-ddvT	pJB861 with a 3.3-kb KpnI-EcoRI fragment carrying *ddvT*	This study
pJB-tonB2	pJB861 with a 1.2-kb NotI-EcoRI fragment carrying *tonB2* from pJB866 including *tonB2*	This study
pJB-tonB2His	pJB861 with a 1.0-kb fragment carrying *tonB2* fused with a His6 tag at the C-terminus	This study
pS-XR	pSEVA225 with a 0.8-kb PCR amplicon carrying *ddvR* and *ligXa* promoter regions	34
pS-ddvT	pSEVA338 with a 3.3-kb KpnI-PstI fragment carrying *ddvT* from pJBddvT	This study
pS-ddvT^{V42A}	pS-ddvT with point mutation in TonB box of *ddvT* (V42A)	This study
pS-ddvT^{T43A}	pS-ddvT with point mutation in TonB box of *ddvT* (T43A)	This study
pS-ddvT$^{V42A\text{-}T43A}$	pS-ddvT with point mutation in TonB box of *ddvT* (V42A, T43A)	This study
pS-tonB1	pSEVA338 with a 0.7-kb fragment carrying *tonB1*	This study
pJB-tonB1	pJB861 with a 0.7-kb NotI-SacI fragment carrying *tonB1* from pS-tonB1	This study
pS-t1-D1	pSEVA338 with a 2.1-kb fragment carrying *tonB1*, *exbB1*, and *exbD1*	This study
pJB-t1-D1	pJB861 with a 2.1-kb NotI-SacI fragment carrying *tonB1*, *exbB1*, and *exbD1* from pS-t1-D1	This study

pS-t1-D12	pSEVA338 with a 2.6-kb fragment carrying *tonB1*, *exbB1*, *exbD1*, and *exbD2*	This study
pJB-t1-D12	pJB861 with a 2.6-kb NotI-SacI fragment carrying *tonB1*, *exbB1*, *exbD1*, and *exbD2* from pS-t1-D12	This study

[a]Nal^r, Sm^r, Km^r, Tet^r and Cm^r, resistance to nalidixic acid, streptomycin, kanamycin, tetracycline and chloramphenicol, respectively.

Supplementary Table 4. Primers used in this study

Target gene	Primer	Sequences (5′ to 3′)
For gene disruption		
pAK01650	Dis_TopF	CGGTACCCGGGGATCATCCGGTTCAGCGCATGT
(*tonB5*)	Dis_TopR	CTGGCAGTTCCGCGTTCT
	Dis_BotF	AGAACGCGGAACTGCCAGTCGAGCGGTGGAAGAGAA
	Dis_BotR	CGACTCTAGAGGATCATCACCCAGTCGGGATGCT
pAK04460	Dis_TopF	CGGTACCCGGGGATCTTCGAGGACTGGGAAGCGT
	Dis_TopR	ACATCGATCCGGCGCGATT
	Dis_BotF	AATCGCGCCGGATCGATGTAAGGCAATCGCTTCGCGCT
	Dis_BotR	CGACTCTAGAGGATCGGACCATGTCATCGTCCA
pAK07650	Dis_TopF	CGGTACCCGGGGATCTCGGCGTCTTCAATCCAC
(*ddvT*)	Dis_TopR	AGGACCGTCCAGAGAACC
	Dis_BotF	GGTTCTCTGGACGGTCCTGGTGGGCGTTGTCGCTTA
	Dis_BotR	CGACTCTAGAGGATCGTGGTGGAAAATCGACGG
pAK14690	Dis_TopF	CGGTACCCGGGGATCCCAGCTGCCCGCATATGT
(*tonB6*)	Dis_TopR	AGATCCTGCACCGCGAGA
	Dis_BotF	TCTCGCGGTGCAGGATCTTCTCATCCTGCGCAATGG
	Dis_BotR	CGACTCTAGAGGATCCCGATCAGCTGCACATCA
pAK34540	Dis_TopF	CGGTACCCGGGGATCAGCGTGAACACGATGCAGGC
(*tonB2*)	Dis_TopR	TGCGCCTTGAAAGCCGGAT
	Dis_BotF	ATCCGGCTTTCAAGGCGCAAAAGCATGATCACGGCCCG
	Dis_BotR	CGACTCTAGAGGATCATCGTTGCGGTGGCCCAT
pAK36940	Dis_TopF	CGGTACCCGGGGATCGAATCATGCGCGCAAGGTTC
(*tonB3*)	Dis_TopR	GGCGTCCAATCCGGTCTTCT
	Dis_BotF	CGACTCTAGAGGATCCGCTCACCGGCATGGATCAT
	Dis_BotR	AGAAGACCGGATTGGACGCCTGCCATAAGTGGAACGGGGC
pAK37490	Dis_TopF	CGGTACCCGGGGATCATACGCCACCTGACGGAC
(*tonB6*)	Dis_TopR	CTTCTCGACGGTATCGCC
	Dis_BotF	GGCGATACCGTCGAGAAGGATAGCCTCTCCGCCTGA
	Dis_BotR	CGACTCTAGAGGATCAGCCCTCTCGACGCTCTT
pAK38050	Dis_TopF	CGGTACCCGGGGATCCCAGCAGCAGGATGAACA
	Dis_TopR	TTTCAGCCTGCTGGCCAT
	Dis_BotF	ATGGCCAGCAGGCTGAAAGTGAACCGGTTCATCCAG
	Dis_BotR	CGACTCTAGAGGATCGCCTGTCACGATCGCAAT
pAK38320	Dis_TopF	CGGTACCCGGGGATCGGGCATCGACACTATCGA
(*ompW*)	Dis_TopR	TCTTCGAAGTCGATCGCG
	Dis_BotF	CGCGATCGACTTCGAAGATATGTCGGTGGCTTCGGT
	Dis_BotR	CGACTCTAGAGGATCCAGACCGCGAAGAATGTG
For confirmation of gene disruption		
SLG_01650	01650_F	AGACGAGGCCCATCAGTT
SLG_04460	04460_F	TGGACGGCATCCCGCAAAA
SLG_07650	07650_F	TGAGGCGTTGCTGCAACTCG
SLG_14690	14690_F	CGACGCCGATACGAATTT
SLG_34540	34540_F	GCGGAATGGTGATGCTGA
SLG_36940	36940_F	GCGGAATGGTGATGCTGA
SLG_37490	37490_F	ACTCAGCGAGGAAGTGCG
SLG_38050	38050_F	AGTAATTGGCGGCATAAGCG
SLG_38320	38320_F	CGCCAAGCCCGATGAGAA

For RT-PCR		
tonB1-exbB1	Forward	CCCGCAAACTGGATCACG
	Reverse	TGCCATGCTGTTCCTGCG
exbB1-exbD1	Forward	AGCGGCGATCAACTCGAA
	Reverse	TATGCGCCTCGGGGAGAT
exbD1-exbD2	Forward	CCTCGTGGACGTCATGCT
	Reverse	TCGACCACCACATAGCGG
Construction of plasmids		
pJB-ddvT	Forward	AAAGGTACCTTCAAACGCCAGCGGGACA
	Reverse	AGGCCGAATTCTTCAGAAGCCGAACCGCA
pJB-tonB2His	Forward	GAAGCTTCGTGGATCATTACAGGACCGGGCACG
	Reverse	CAGGATATCTGGATCTCAGTGGTGGTGGTGGTGGTGGGTCTCGATCGTCTTG
pJB-tonB1	Forward	GCGGCCGCGCGAATTCGCTTTAGAGCTAAGGAGT
	Reverse	TACCGAGCTCGAATTTCATTCAGGCAGACGCCA
pJB-t1-D1	Forward	GCCTAGGCCGCGGCCGCGGCTACGGCCCAGAAGTAG
	Reverse	TACCGAGCTCGAATTTCAGAGGCGACGGACGGT
pJB-t1-D12	Forward	GCCTAGGCCGCGGCCGCCGCCCAGATTTAGGAGACCAG
	Reverse	TACCGAGCTCGAATTTCAGAACACGCCGCCGTA
Mutation of *ddvT*		
pS-ddvT^{V42A}	Forward	GCTACCGGGTCGCGCGTGGCCCGAAG
	Reverse	GATGATGTCCTGAGCCTGTT
pS-ddvT^{T43A}	Forward	GCTGGGTCGCGCGTGGCCCGAAG
	Reverse	GACGATGATGTCCTGAGCCT
pS-ddvT$^{V42A\text{-}T43A}$	Forward	GCTGCTGGGTCGCGCGTGGCCCGAAG
	Reverse	GATGATGTCCTGAGCCTGTT

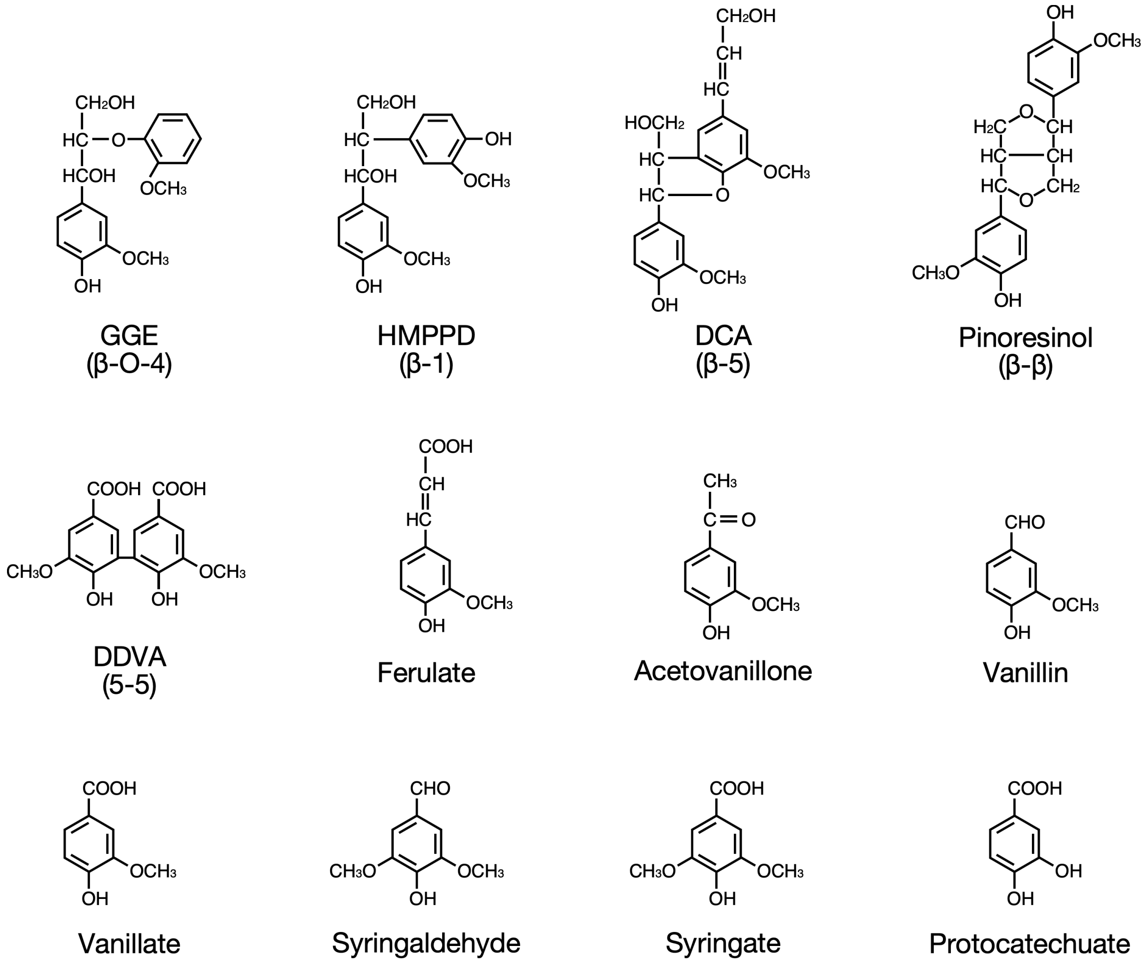

Supplementary Figure 1. Chemical structures of lignin-derived aromatic compounds used in this study. GGE, guaiacylglycerol-β-guaiacyl ether; HMPPD, 1,2-bis(4-hydroxy-3-methoxyphenyl)-propane-1,3-diol; DCA, dehydrodiconiferyl alcohol; DDVA, 5,5′-dehydrodivanillate.

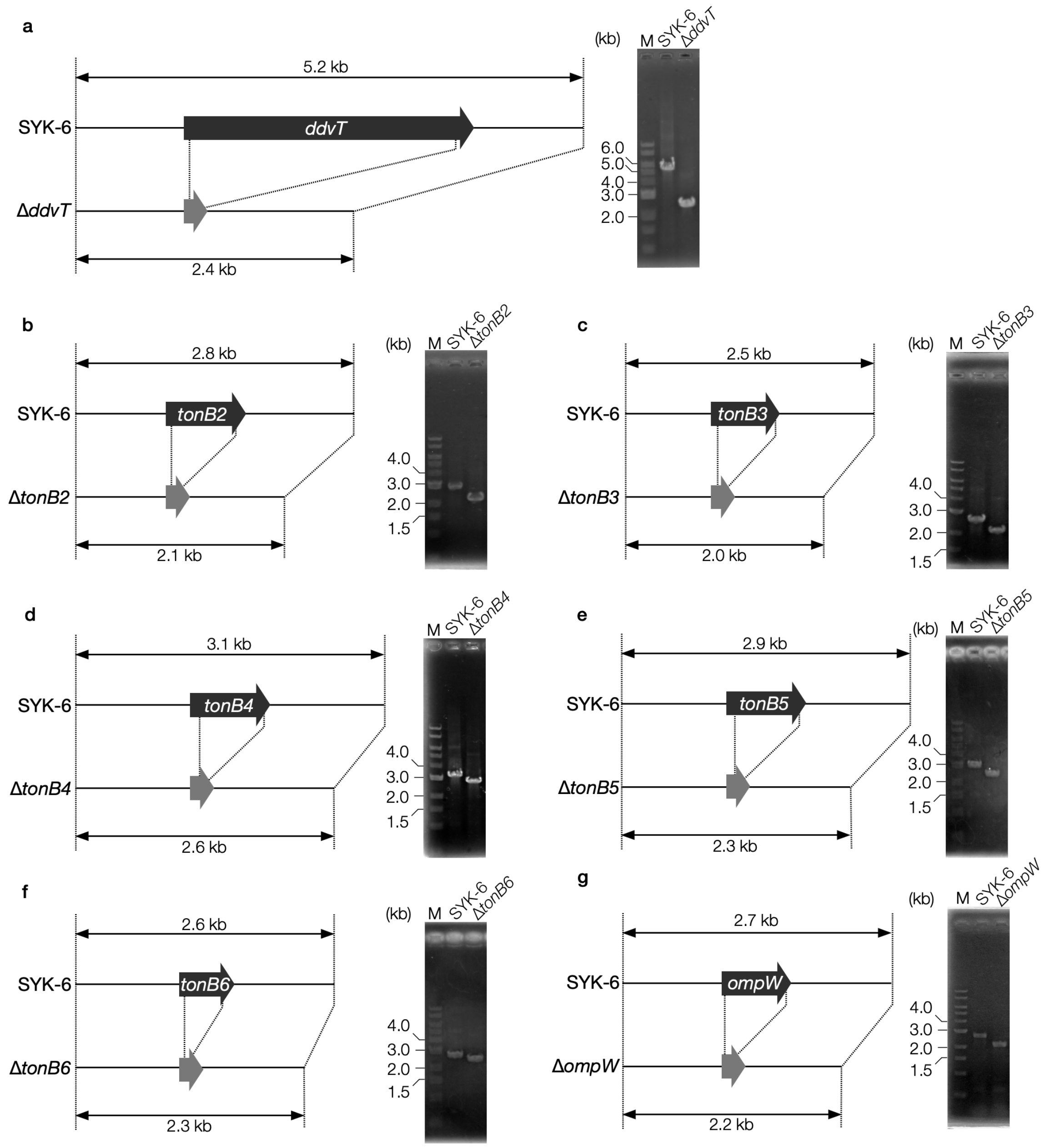

Supplementary Figure 2. Construction of mutants. Schematic representations and colony PCR analyses of the disruption of *ddvT* (a), *tonB2* (b), *tonB3* (c), *tonB4* (d), *tonB5* (e), *tonB6* (f), *ompW* (g), SLG_04460 (h) and SLG_38050 (i). The primer pairs used for colony PCR analyses were shown in Supplementary Table 4. M, molecular size markers.

h

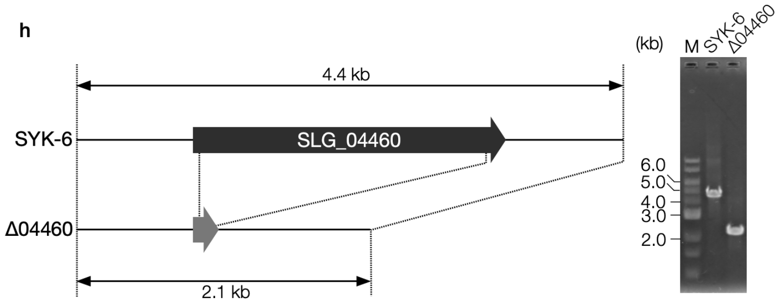

i

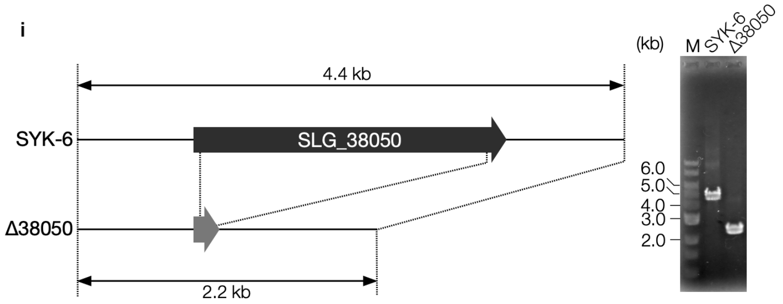

Supplementary Figure 2. *–continued.*

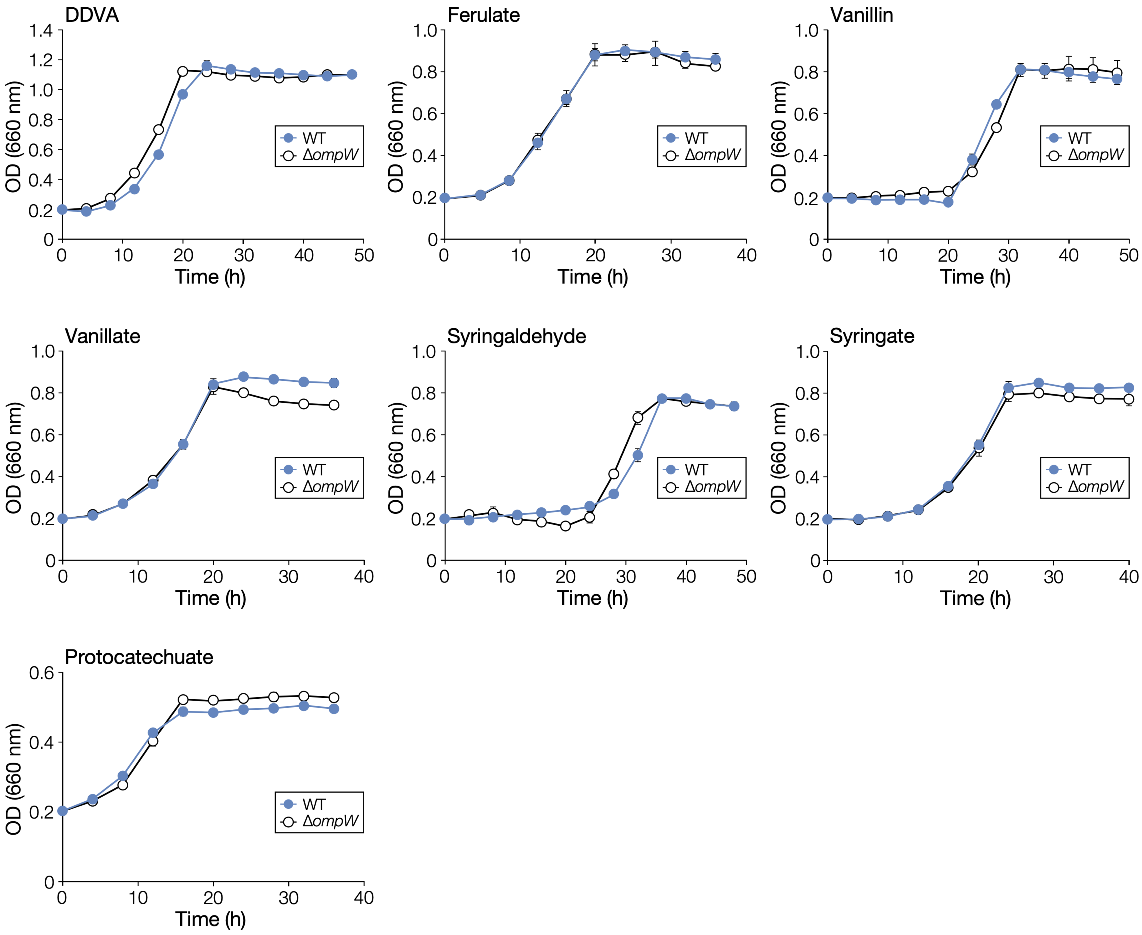

Supplementary Figure 3. Growth of an *ompW* mutant on lignin-derived aromatic compounds. Growth of cells of SYK-6 and Δ*ompW* (SLG_38520) in Wx medium containing 5 mM DDVA, FA, VN, VA, SN, SA and PCA, respectively. Cell growth was monitored by measuring the OD_{660}. Each value is the average ± the standard deviation of n = 3 independent experiments.

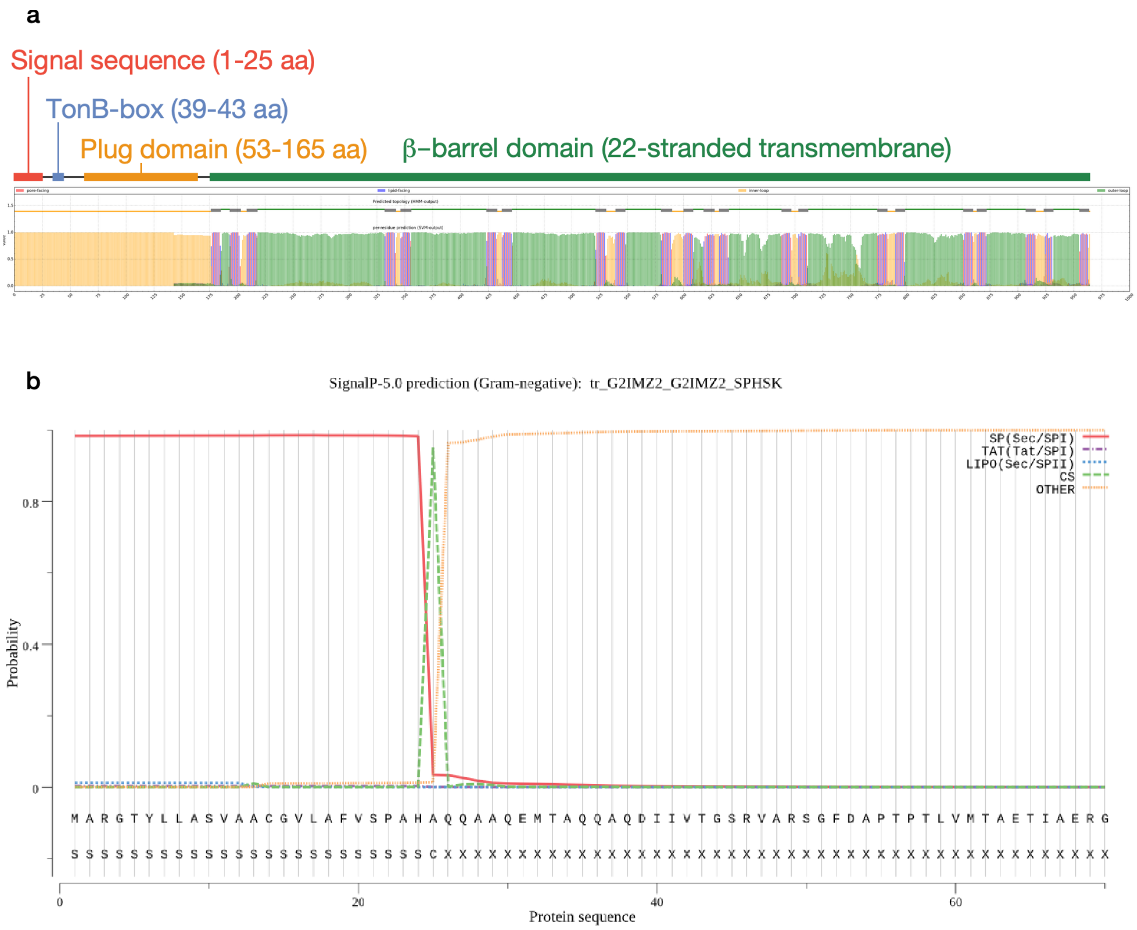

Supplementary Figure 4. Prediction of the transmembrane segments and signal sequence of DdvT. a, Transmembrane segments of DdvT as predicted by BOCTOPUS2[40]. **b,** The signal sequence of DdvT (Met1 to Ala25) as predicted by SignalP-5.0[41].

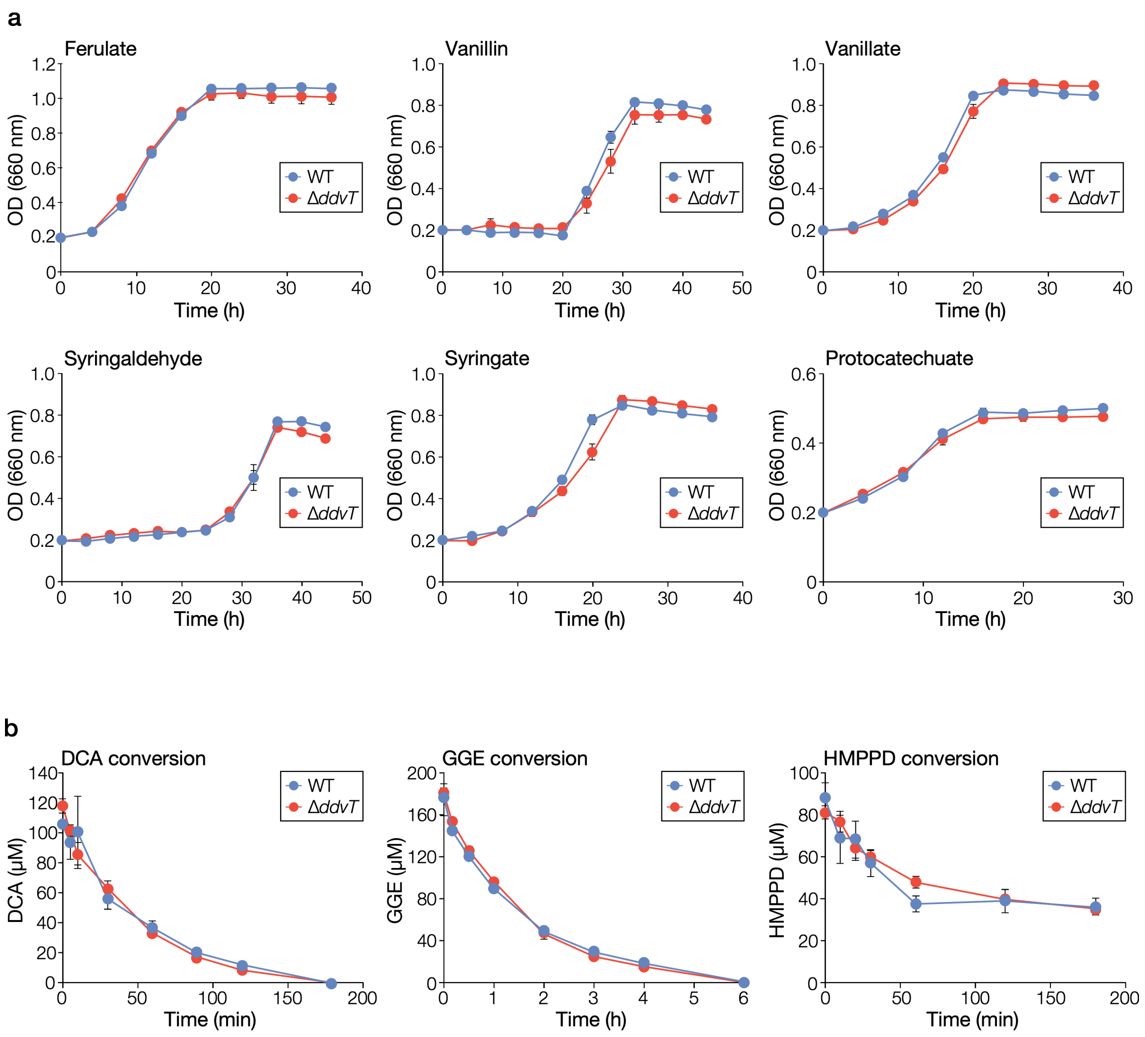

Supplementary Figure 5. Characterisation of the *ddvT* mutant. a, Growth of cells of SYK-6 and Δ*ddvT* in Wx medium containing 5 mM FA, VN, VA, SN, SA and PCA, respectively. Cell growth was monitored by measuring the OD_{660}. **b,** Conversion of lignin-derived biaryls by resting cells of Δ*ddvT*. Cells of SYK-6 and Δ*ddvT* were incubated with 200 μM DCA, 200 μM GGE and 100 μM HMPPD, respectively. Portions of the reaction mixtures were collected and the amount of substrate was measured by HPLC. Each value is the average ± the standard deviation of $n = 3$ independent experiments.

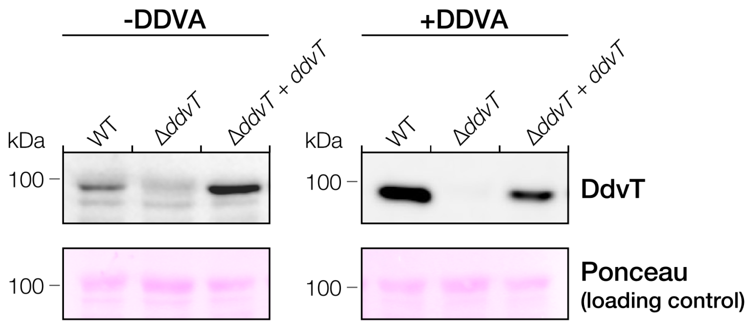

Supplementary Figure 6. Expression level of *ddvT* in Δ*ddvT* cells harbouring pS-ddvT. Western blot analysis using anti-DdvT antibodies was performed against total membrane fractions (10 μg of protein) obtained from the cells of SYK-6(pSEVA338), Δ*ddvT*(pSEVA338) and Δ*ddvT*(pS-ddvT) grown in LB containing 0.5 mM *m*-toluate with or without 1 mM DDVA. Ponceau S staining is shown as loading control.

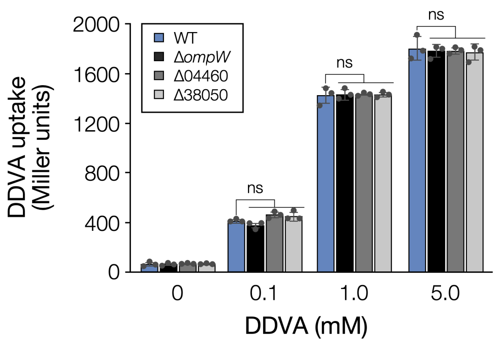

Supplementary Figure 7. DDVA uptake by Δ*ompW*, Δ04460 and Δ38050 cells. The β-galactosidase activities of cells of SYK-6(pS-XR), Δ*ompW*(pS-XR), Δ04460(pS-XR) and Δ38050(pS-XR) incubated in Wx-SEMP with or without DDVA (0.1 mM, 1.0 mM and 5.0 mM) were measured. Each value is the average ± the standard deviation of n = 3 independent experiments. ns, $P > 0.05$ (one-way ANOVA with Dunnett's multiple comparisons post-test).

TonB box [X-X-X-T]

```
SLG_32710  AAWAQDTA--GTGQSDTAGSPDLAQDDQPDISIPGGGEIIVTGRRSG-------------  65
SLG_30030  EEISQTVE--ITAGGTTTLNFDLQQTGV--TQASSGNDIVVTGTVRE--------GDARA  216
SLG_28010  A--QTTTV--DPA----------SEDG-------AGEEIVVSGYRR---------SIEEA  60
SLG_07450  A---QQTV--APEDG---------AEE--------ADEIVITGSILS--------AQQDS  57
SLG_11490  ETKSETVT--VPAQGRVTLNFGLSGYG--------PDDILVVGQAA---------NLSSS  146
SLG_37500  ---AQPVH------EL---PPV--E----VEAGPGPAKIATAPLT---------------  50
SLG_17080  ---AQEPQ------VE---RDD--PDP--RERGSARRDIVVTGQS-------------L-  56
SLG_34070  ---AQDRA------TQ---PPD--TGAGHGHDADHPEDIVITAII-------------P-  62
SLG_17580  ---AQTLDEKETEQTDGTMLPP--VGAGTSGEFGDEEEIVVTGAR-------------ER  68
SLG_28800  ---AQD-A------A------------PEDANETTGDAIIVTGST-------------RA  48
SLG_36280  ---AQS-----------------------TGAIDFEDAIIVTGSRS---------SDVGG  59
SLG_16980  ---AQT-G------TP----------TGTGTRGSAPDDILVIGTL---------NTDQAR  50
SLG_34550  ---AQA-Q------TG------------ADTQQPALGGVTVTDTAIDDTRETRVETPKAT  64
SLG_04380  ---AQD-N-------------------DGLYLLNRLNRIVVTATR-------------TP  53
SLG_10790  ----------------------------EAGAPAIASDIVVTANR-------------TE  44
SLG_31170  ---AQD---------------------SPADEADGGGEIVVTATR-------------KS  51
SLG_17010  ---AQQ-A------P----------LPADAGESDGADTIIVTGRVQRLYRAEQTTVGKAA  64
SLG_07950  ---AQQPQ------AD-----------TAVSDEGATDAIIVTARR-------------RA  51
SLG_15260  ---VLA-Q------DT-----------AAAPREQGIQDIVVTATR-------------QA  49
SLG_28170  ---AMA-Q------EE-----------A---SRAMPDDIIVTATK-------------MA  42
SLG_12400  ---AQG-E------ST-----------PQDNPGADPTDIIVTAQF-------------RE  49
SLG_09260  ---AQG-T------AT-----------AAA-DENDDDVIVVTAQM-------------RE  61
SLG_18520  ---ALA-Q------ET-----------PQA-DNVGTDDIIVTAQF-------------RA  46
SLG_38050  ---GQQ-A------ET-----------PAP-QDDSNADIVVTAQF-------------RS  53
SLG_18600  ---AQE-T------EA-----------RQE-EAAGLADIVVTAQY-------------RA  52
SLG_27640  ---ANG-Q------TA-----------QTG-SEASETDIVVTAQF-------------RA  45
SLG_27360  ---AQD-I----------------------APADQPMEIIVTGEK-------------AS  43
SLG_20440  ---AQT--------------------AGDSASDDDAAAIVVTARR-------------RE  55
SLG_30910  ---AQ---------------------ARAEAEPADADTIIVTAQL-------------RE  41
SLG_27890  ---AQG-A------AAG----A--PAPQEERVDAAEGDIVVTAQR-------------RS  57
SLG_37710  ---AQD-G------SAG----S--LPQDAADDPDNAGDIIVTATR-------------RA  75
SLG_36760  ---AQD-S------TPP----Q--DDPTVPQAESRAGEIVVTARR-------------RS  71
SLG_24600  ---AQG-T------QPQ----G-----STTTADTYGDEIIVTAQK-------------RE  46
SLG_08200  ---AQD-N------SAP----D--E-------PYDGGEIIVTAQK-------------SA  44
SLG_28640  ---AQS------------------DAAPPAEEGQGLEEIIVTAQR-------------VQ  47
SLG_14310  ---AQG-S----------------ASAEIETGEQGLQDIVVTAQR-------------RS  50
SLG_11290  ---LHA-Q------EAP----D--E--AAGANDASTEEIVVTAQF-------------RR  54
SLG_18440  ------------------------------------------------------------  0
SLG_18950  ---AQA-V------EGQ----D--ESSSGADQSNGIQDIVVTASR-------------RA  58
SLG_18450  ---AQE---------TD----P----PPPDLTGSVIQDIVVTASR-------------RS  54
SLG_18820  ---AQD---------AG----Q--SSPQDTASSTGIADIIVTASR-------------RA  65
SLG_04340  -------------------------PALAQAPADDPQDIIITAQR-------------AN  57
SLG_10860  ---AAE---------------A--TASDEEATEDARKAILVYGHR-------------SD  51
SLG_28750  -------Q------AS-----G--DAAVAPQEEEVDQAIIVTGTR-------------IG  71
SLG_19710  -------R------ES-----S--FTAKNDRSENGENEIIVTGSH-------------LK  127
SLG_26880  -------R------AR-----P--HAARQD--DPGPGEIVVTGTR-------------IR  128
SLG_04460  KRRVPE-Q-------------------PLPTVSDDETPIIVTASK-------------MD  141
SLG_25590  ---------------------------LLAEAPPSPEDIIVLGRG-------------LD  47
SLG_30670  ---------------------------VPAMAQAIQETIIVTATG-------------AP  40
SLG_17060  SDSAIA-Q------PT-----------PGDAMSDTMGEIVVTGTN-------------LR  53
SLG_11850  ---AQT----------------------AEAEADAPDDIVITGSL-------------IS  60
SLG_31810  ---AQD-T------VT-----------PQAAAAPEDQAIVITGSR-------------IA  57
SLG_06600  ---AQE-P------QE-----------AQADTAGQNEAIVITGSR-------------IA  51
SLG_05610  -------V------HA-----------QEAPQAAEEQAIVVTGSR-------------IV  51
SLG_35890  -------A------QD-----------ETGPQASQDQAIVITGSR-------------IV  60
SLG_25090  ----RP-A------RA-----P--PSLAQVGAAPAEETIILTGSR-------------IR  143
SLG_25150  ----IP--------GG-----P--AWAQDEDGAASTSSIVVTGSR-------------IR  44
SLG_02360  ---AQ----------E-----P-----QGADDAETGDVIIITGSL-------------IK  53
SLG_18250  ---QTE-E------T-------------APQAGGSGEEIVITGSR-------------LV  61
SLG_34920  ---AQ--E------TQ-----P-----QDGEAAADQGLIVITGSR-------------IK  61
SLG_02250  ---AQD-N------IA-----P-----QAASEGEDGAAILVTGTR-------------IK  35
SLG_28190  ---AQE-S------AA-----P-----QGASEEADPGTVIVTGSR-------------IK  81
SLG_26390  ---AVG-G------ST-----N--SAAAGASIEGGNNDIVVTGSR-------------VI  70
SLG_27490  ---VAA-E------AA-----P--ESAAAGSAAAPAQDIVITGSR-------------IQ  69
SLG_31220  ---QQG-E------GI-----E--VAEASDNGAASNQDLVITGSR-------------I-  72
SLG_31370  ----QP-E------AV-----E--DAN-VADETENKQDLVITGTR-------------L-  59
SLG_27320  ---QVE-Q------A------------EISS-QATAADIIVTASR-------------AA  61
SLG_34990  ---AAG-Q------DA-----Q-----SPQD-MPPEGEIIVTGSR-------------AA  59
SLG_14220  ---T---A------GA-----Q-----GIDAEEGGSDAIVITGSR-------------IA  45
DdvT       ---QA----------------------AQEMTAQQAQDIIVTGSR-------------VA  48
SLG_27590  ---AQE-S------AT-----D-----VPAESGTSNEAIIVTGSR-------------IV  58
SLG_28830  ---AAE-A------PA-----P-----APEAPDAESEAIVITGSR-------------IA  150
SLG_09330  ---QD----------T-----A-----VPQEEPVSEEAIVVTGSR-------------IA  66
SLG_25080  ---AA----------T-----S-----STADNAPSTEAIVVTGSR-------------LA  30
```

Supplementary Figure 8. TonB box-like sequences conserved among the putative SYK-6 TBDRs. A multiple alignment of the N-terminal amino acid sequences deduced from the putative SYK-6 TBDR genes was constructed using the Clustal Omega program[42].

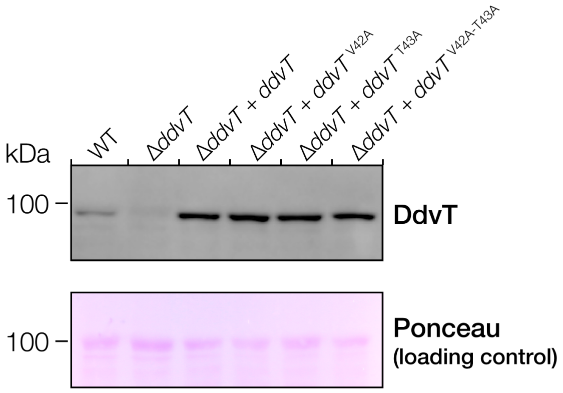

Supplementary Figure 9. Expression of TonB box mutated *ddvT* in Δ*ddvT*. Western blot analysis using anti-DdvT antibodies was performed against total membrane fractions (10 μg of protein) obtained from cells of SYK-6(pSEVA338), Δ*ddvT*(pSEVA338), Δ*ddvT*(pS-ddvT), Δ*ddvT*(pS-ddvT^{V42A}), Δ*ddvT*(pS-ddvT^{T43A}) and Δ*ddvT*(pS-ddvT$^{V42A–T43A}$) grown in LB containing 0.5 mM *m*-toluate. Ponceau S staining is shown as loading control.

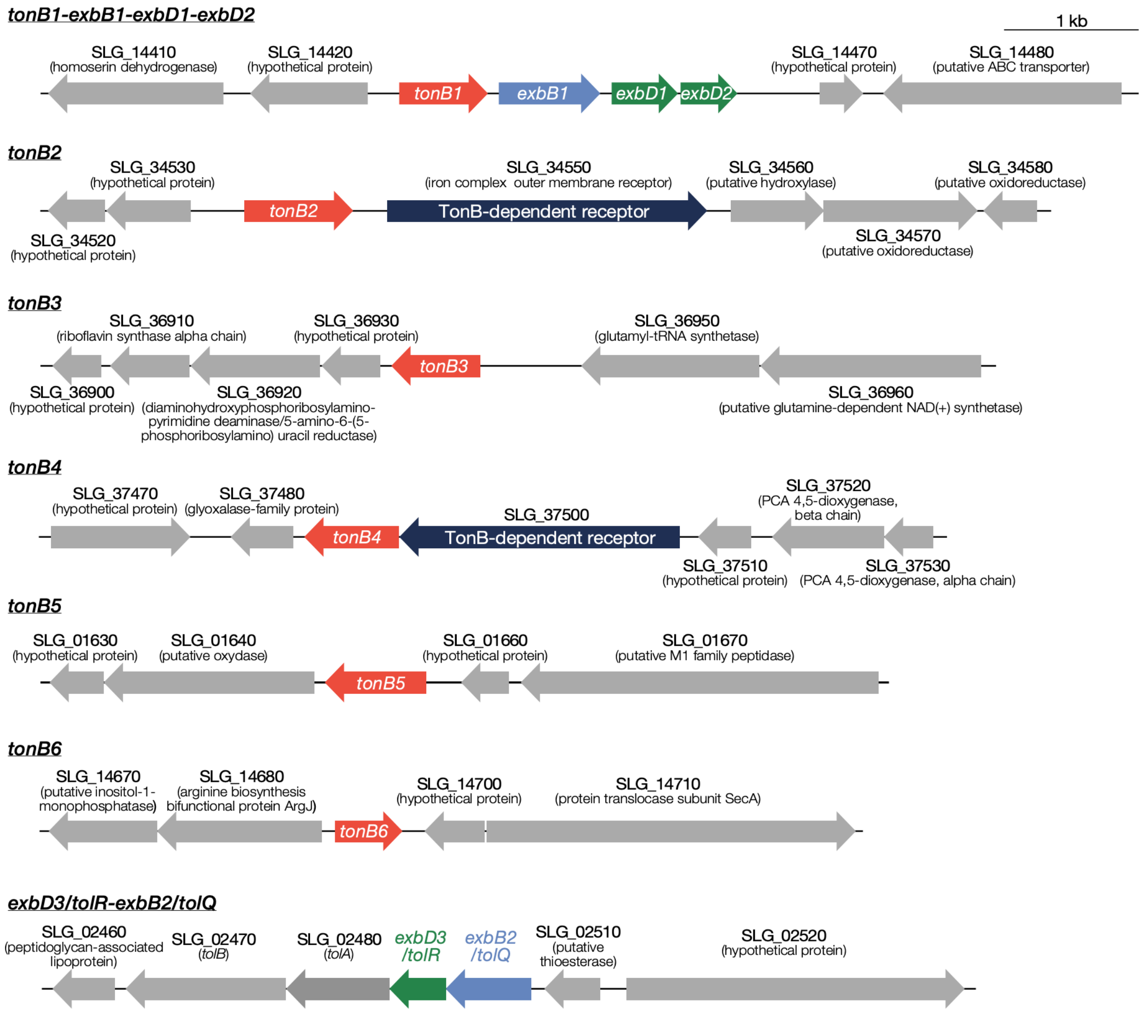

Supplementary Figure 10. Organisation of the putative *tonB, exbB* and *exbD* genes in the SYK-6 genome.

```
                                              Transmembrane domain
TonB1    ----------MAYADHSQS----------SSRTI-S--IV-VVAIIHV------ILGYAFVT-   32
TonB2    -MNAYFDPAFKAHTPPAEAA--SERSAYRRSRG--SDSVATVGALVFGLATVGAFAFM--   53
TonB3    ---------------MSDMS--VPRSTYGSRRS--PAAMAAALALN-----GGLFALLIA   36
TonB4    -----MVP---PGP---SLPARSACRTFGGRGGGRAAAMVAALAVHGLVA---LLVLLAP   46
TonB5    ---------MLAGERYGGV--------SVRARK--LGPLALTLALHALVIALLLFHRSMP   41
TonB6    -------MN-----RK----------SADLLNA--LG-GVITIAS------LLAFAWSQI   29
EcTonB   ------------------MTLDLPRRFPWPT----LLSVCIHGAVV------AGLLYTSV   32
PaTonB   AVEEVLIP-YAHGSDPEDVPGEPPKSRWWLS-S--GAAVAMHVAII------GALVWVMP  110

TonB1    -GLGIQYVKKA---AEQLNVIDVAEEPPPPEEEPPPPPP-----PPPDMPPPPPP--PPS   81
TonB2    HPSFVRK-APRT--PTIVTMM-----------ELPDDPPPAPPEQPPAPETPPPPSAQVV   99
TonB3    LPVAVQV-IPAD--PPI-RIR-----------HVPLDPLPSSEPEPQKTESP-RNVVQPV   80
TonB4    SSRPVASGG------EAIRVFALPRLAEPLPRHETRHVEGVRDAAP------------PG   88
TonB5    IPRTVEQ--------SLTTIFLPASKPEQADASKARTQR---QAKEAASEAPKPA-----   85
TonB6    AVDGLTRRAGR---VVHLAVQDLT------------------------------------   50
EcTonB   HQ-VIELPAPA--QPISVTMVTPADLEPPQAVQPPPEPVVEPEPE----PEPIPE---PP   82
PaTonB   TPAELNLGHGELPKTMQVNFVQLEKKAEP----TPQPPAAAPEPTPPKIEEPKPE---PP  163
             :              .   Proline rich region

TonB1    APPPVISLPSQAPVLAPPPPP-IPRPP------------------------APPAPPAPP  116
TonB2    APVPLVVLPER-P------APLAPAVA------------------------T---PPAPP  125
TonB3    IPHPVQPDPVR-V------EPLVPLDTG--------------PQIALSEFRLPITPAEPI  119
TonB4    AERSLVPL--HGDKAKPPAG----SFEGSVPLPALVPAVP--SAPAVSMMSAPAV----P  136
TonB5    ------TSPPEPPKPDPSPAPVLPNYLTMTSAE--------FAAADISRMPS--------  123
TonB6    -----INLGMAGSAITPGPPPVIMLPP------------------------G--------   73
EcTonB   KEAPVVIEKPK-PKPKPKPKPVKKVQEQPK--RDVKPV--------------ESR----P  121
PaTonB   KPKP--VEKPK-PKPKPKPKPVENAIPKAKPKPEPKPKPEPEPSTEASSQPSPSSAAPPP  220

TonB1    PPPVVSKAAG-----------A---------RGNPANWITND------DYPSRALRDEAQ  150
TonB2    A-PVRAA------PP-----PAPRGPQDMGDISARMISARPP------AYPLASRREKEE  167
TonB3    A-PIESP------AE-----PV----LHKARPDPRFADAFRP------AYPAALRRDGLE  157
TonB4    S---PKTTPS-----------AD--EPSRLQAYQQILWAQIA--------AHRPRATRMA  172
TonB5    ----HSRSRGSSTQEADSEAAYGPGEGPGGARLYEADWYRKPSDAELAGYLPANR--PRR  177
TonB6    ------SKAP-----------R---------GRGPATWITND------DYPMEALRNEQS  101
EcTonB   ASPFENTAPARLTSS-TATAATSKPVT-SVASGPRALSRNQP------QYPARAQALRIE  173
PaTonB   APTVGQSTPGAQTAPSGSQGPAGLPSGSLNDSDIKPLRMDPP------VYPRMAQARGIE  274
                                     C-terminal domain
TonB1    GTVAIEWEINEQGRVENCRVTSSSGN-RDLDEAACSLIVRRGRYSPALDQAGNPIRTKDR  209
TonB2    GTVMLSVLLAIDGHVAEIAIARSSGF-PRLDRAALD-AVSDWRWSPMTRDGEP-VMVRGV  224
TonB3    GSVTVRVTIDPRGRVTDVEMVSASNP-VFFEETRRQ-ALRFWRFVPATRDGVA-VQSVQT  214
TonB4    GTVRVRFQLDHGGALISCEVTKSSGL-MLLDGIALR-SVRQASPFPAPPEELAEDALQFE  230
TonB5    GWGLIACRTIEDYRVDDCRTLGESPLGSGFGRAVRE-AAWQFRVLPPRINGKPMVGSWVR  236
TonB6    GVSQIAWTIEPDGRAGQCHVVRGSGH-AVLDEAARRLILRNGRYEPARDAQGRAIRAVDQ  160
EcTonB   GQVKVKFDVTPDGRVDNVQILSAKPA-NMFEREVKN-AMRRWRYEPGKPGSGI-VVNI--  228
PaTonB   GRVKVLFTITSDGRIDDIQVLESVPS-RMFDREVRQ-AMAKWRFEPRVSGGKI-VARQAT  331
         *   :           .            :               *

TonB1    RRVVWRLPE---------------------------------  218
TonB2    VTIPFILQRGDPGRHHRRGKHDHGPDPRDRPLSDGQIKTIET  266
TonB3    MTVHFRLEN---------------------------------  223
TonB4    VPVTFGRERPD-------------------------------  241
TonB5    IRIEYGEREGP-------G-----------------------  248
TonB6    RRVVWRLAD---------------------------------  169
EcTonB   ---LFKINGTTEIQ----------------------------  239
PaTonB   KMFFFKIEKRR-------------------------------  342
             :
```

Supplementary Figure 11. Multiple alignment of the deduced amino acid sequences of the SYK-6 *tonB* genes with those of the known *tonB* genes. A multiple alignment was constructed using the Clustal Omega program[42]. Transmembrane (TM) domains were predicted by TMHMM 2.0[43]. The TM domains and the proline-rich regions are highlighted in red and cyan, respectively. The C-terminal amino acid sequences of TonB from *P. aeruginosa* (*Pa*TonB, AAG08916) and *E. coli* (*Ec*TonB, AAB59066) predicted to interact with the TonB box are highlighted in black[44].

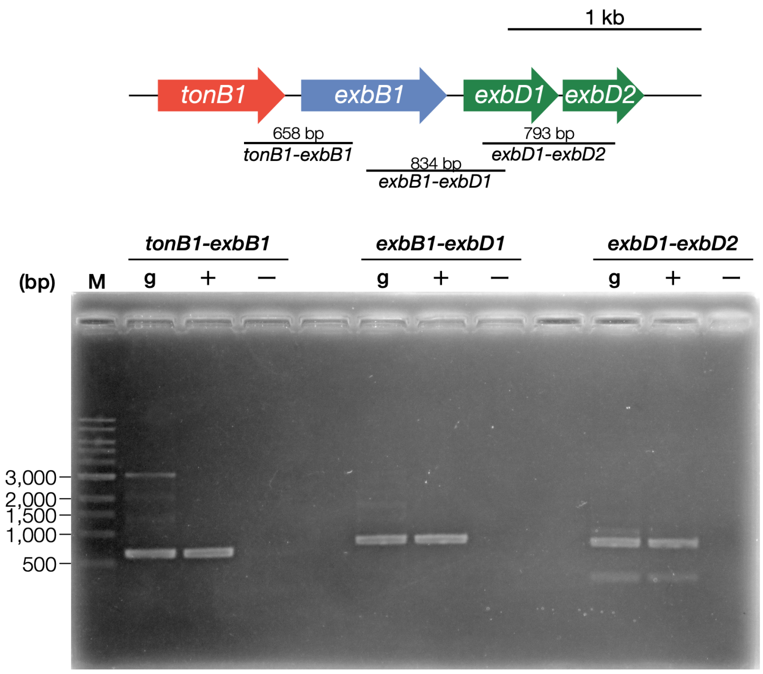

Supplementary Figure 12. RT-PCR analysis of the *tonB1* gene cluster. Total RNA used for cDNA synthesis was isolated from SYK-6 cells grown in Wx-SEMP. The positions of the primer pairs are indicated below the gene map (Supplementary Table 4). Lanes: M, molecular size markers; g, control PCR with the SYK-6 genomic DNA; '+' and '–', RT-PCR with and without reverse transcriptase, respectively.

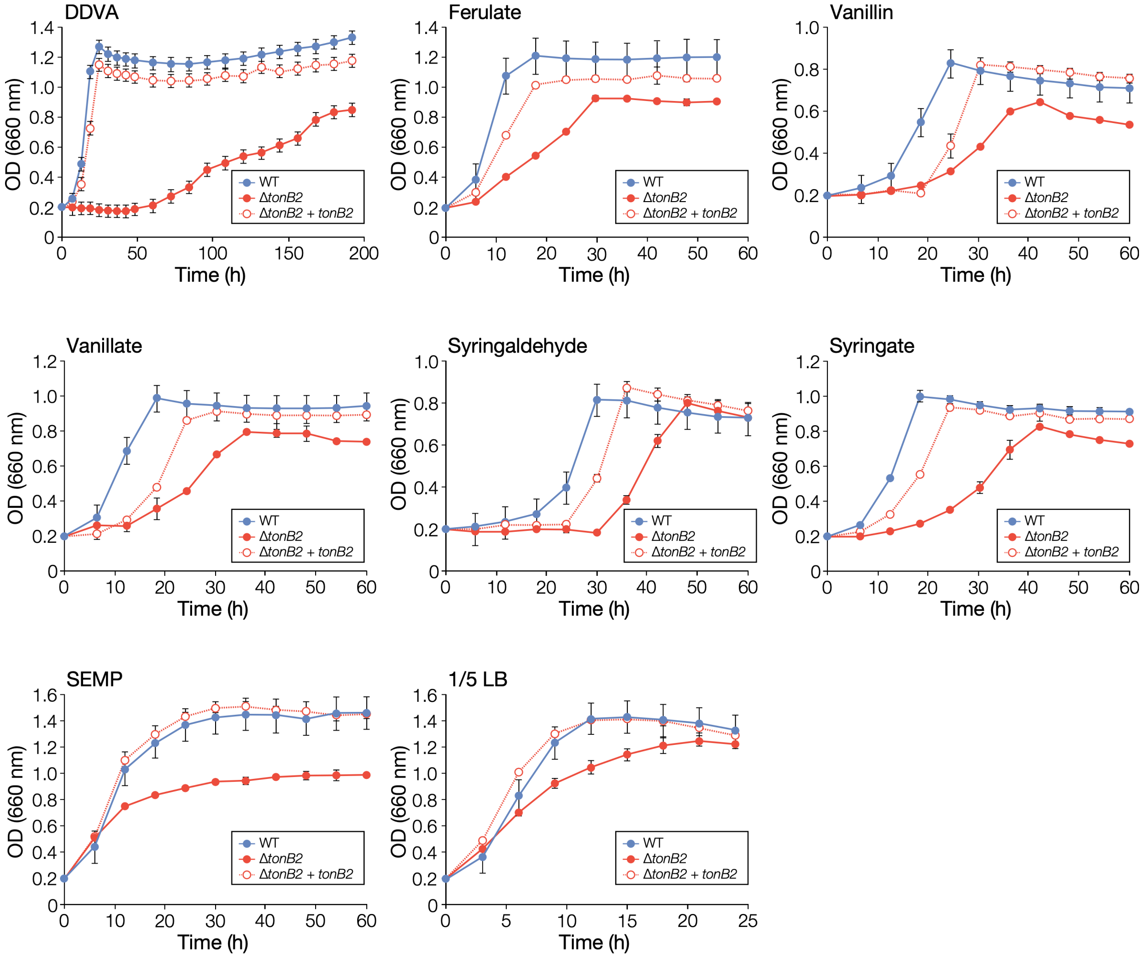

Supplementary Figure 13. Growth of a *tonB2* mutant on lignin-derived aromatic compounds. Cells of SYK-6(pJB861[vector]), Δ*tonB2*(pJB861) and Δ*tonB2*(pJB-tonB2) were cultured in diluted LB, Wx-SEMP or Wx medium containing 5 mM DDVA, FA, VN, VA, SN and SA, respectively. Cell growth was monitored by measuring the OD_{660}. Each value is the average ± the standard deviation of n = 3 independent experiments.

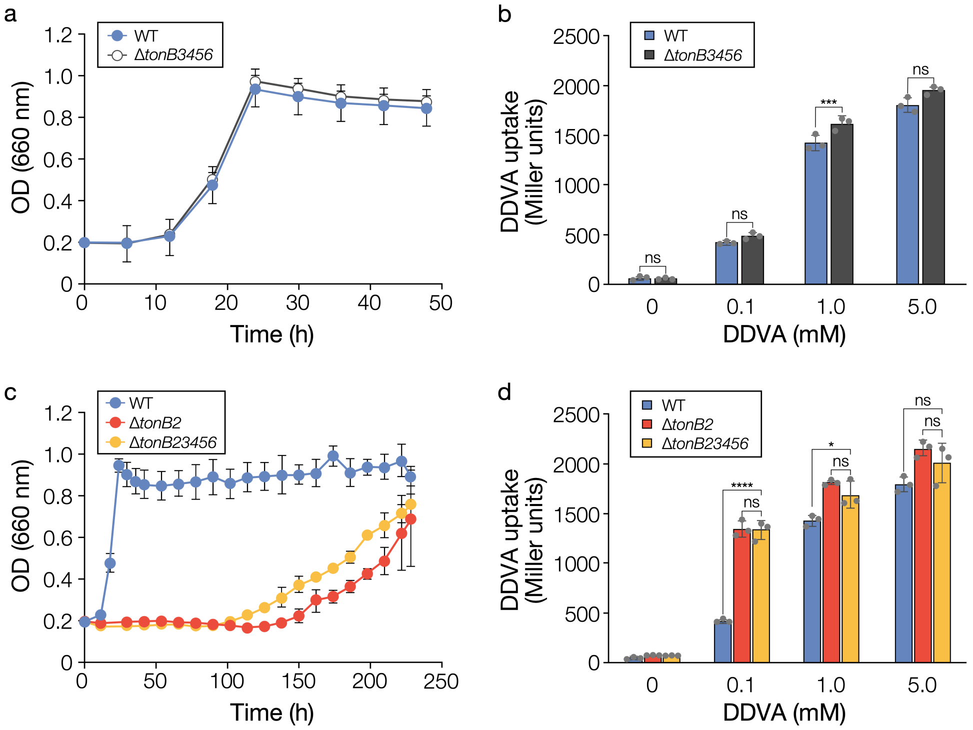

Supplementary Figure 14. Characterisation of *tonB* multiple mutants. a, Growth of a *tonB3 tonB4 tonB5 tonB6* quadruple mutant (Δ*tonB3456*) on DDVA. Cells of SYK-6 and Δ*tonB3456* were cultured in Wx medium containing 5 mM DDVA. Cell growth was monitored by measuring the OD_{660}. **b,** DDVA uptake by Δ*tonB3456* cells. The β-galactosidase activity of cells of SYK-6(pS-XR) and Δ*tonB3456*(pS-XR) incubated in Wx medium containing SEMP with or without DDVA (0.1 mM, 1.0 mM and 5.0 mM) were measured. **c,** Growth of a *tonB2 tonB3 tonB4 tonB5 tonB6* quintuple mutant (Δ*tonB23456*) on DDVA. Cells of SYK-6, Δ*tonB2* and Δ*tonB23456* were cultured in Wx medium containing 5 mM DDVA. Cell growth was monitored by measuring the OD_{660}. **d,** DDVA uptake by Δ*tonB23456* cells. The β-galactosidase activity of cells of SYK-6(pS-XR), Δ*tonB2*(pS-XR) and Δ*tonB23456*(pS-XR) incubated in Wx medium containing SEMP with or without DDVA (0.1 mM, 1.0 mM and 5.0 mM) were measured. Each value is the average ± the standard deviation of $n = 3$ independent experiments. ns, $P > 0.05$, *, $P < 0.05$, ***, $P < 0.001$, ****, $P < 0.0001$ (one-way ANOVA with Dunnett's multiple comparisons post-test).

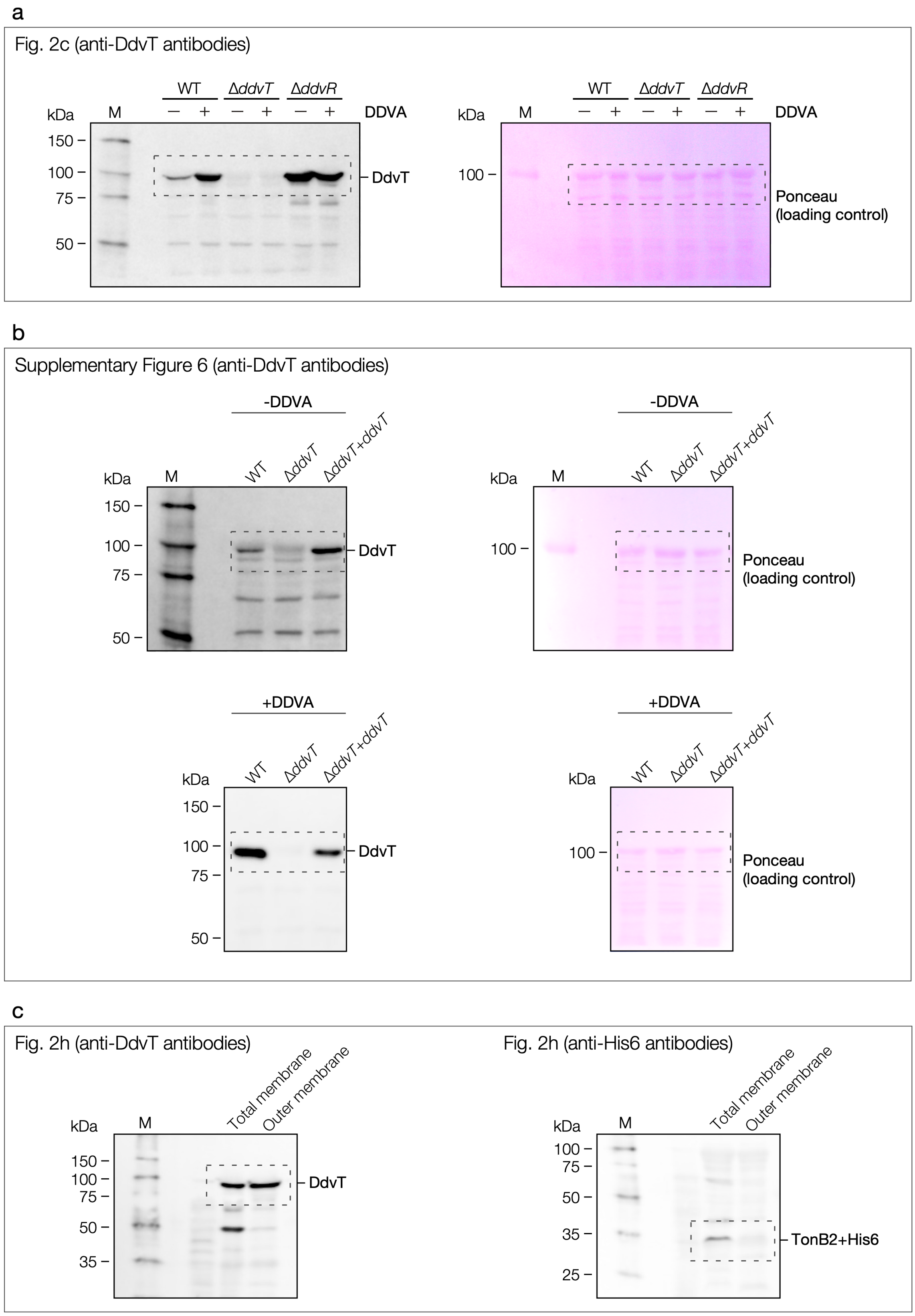

Supplementary Figure 15. Uncropped western blot and ponceau staining images shown in Fig. 2c (a), Supplementary Figure 6 (b), Fig. 2h (c) and Supplementary Figure 9 (d).

d

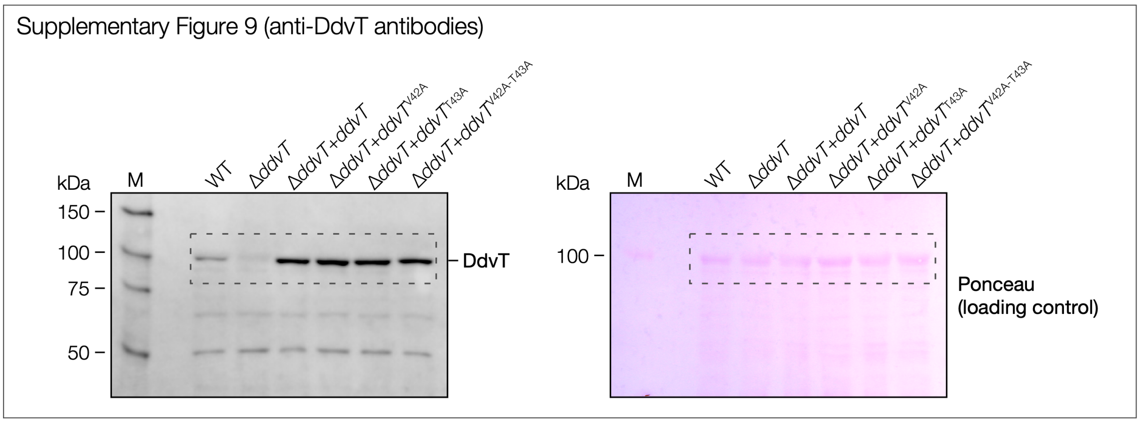

Supplementary Figure 15. *–continued.*

Supplementary References

1 Ravnum, S. & Andersson, D. I. Vitamin B12 repression of the *btuB* gene in *Salmonella typhimurium* is mediated via a translational control which requires leader and coding sequences. *Mol Microbiol* **23**, 35-42, doi:10.1046/j.1365-2958.1997.1761543.x (1997).

2 Heller, K., Mann, B. J. & Kadner, R. J. Cloning and expression of the gene for the vitamin B12 receptor protein in the outer membrane of *Escherichia coli*. *J Bacteriol* **161**, 896-903 (1985).

3 Menikpurage, I. P., Barraza, D., Meléndez, A. B., Strebe, S. & Mera, P. E. The B12 receptor BtuB alters the membrane integrity of *Caulobacter crescentus*. *Microbiology* **165**, 311-323, doi:10.1099/mic.0.000753 (2019).

4 Brickman, T. J. & Armstrong, S. K. Essential role of the iron-regulated outer membrane receptor FauA in alcaligin siderophore-mediated iron uptake in *Bordetella* species. *J Bacteriol* **181**, 5958-5966 (1999).

5 Koebnik, R., Hantke, K. & Braun, V. The TonB-dependent ferrichrome receptor FcuA of *Yersinia enterocolitica*: evidence against a strict co-evolution of receptor structure and substrate specificity. *Mol Microbiol* **7**, 383-393, doi:10.1111/j.1365-2958.1993.tb01130.x (1993).

6 Buchanan, S. K. *et al.* Crystal structure of the outer membrane active transporter FepA from *Escherichia coli*. *Nat Struct Biol* **6**, 56-63, doi:10.1038/4931 (1999).

7 Killmann, H., Herrmann, C., Torun, A., Jung, G. & Braun, V. TonB of *Escherichia coli* activates FhuA through interaction with the β-barrel. *Microbiology* **148**, 3497-3509, doi:10.1099/00221287-148-11-3497 (2002).

8 Sauer, M., Hantke, K. & Braun, V. Ferric-coprogen receptor FhuE of *Escherichia coli*: processing and sequence common to all TonB-dependent outer membrane receptor proteins. *J Bacteriol* **169**, 2044-2049 (1987).

9 Llamas, M. A. *et al.* The heterologous siderophores ferrioxamine B and ferrichrome activate signaling pathways in *Pseudomonas aeruginosa*. *J Bacteriol* **188**, 1882-1891, doi:10.1128/JB.188.5.1882-1891.2006 (2006).

10 Ankenbauer, R. G. & Quan, H. N. FptA, the Fe(III)-pyochelin receptor of *Pseudomonas aeruginosa*: a phenolate siderophore receptor homologous to hydroxamate siderophore receptors. *J Bacteriol* **176**, 307-319, doi:10.1128/jb.176.2.307-319.1994 (1994).

11 Folschweiller, N. *et al.* The pyoverdin receptor FpvA, a TonB-dependent receptor involved in iron uptake by *Pseudomonas aeruginosa* (review). *Mol Membr Biol* **17**, 123-133, doi:10.1080/09687680050197356 (2000).

12 Beucher, M. & Sparling, P. F. Cloning, sequencing, and characterization of the gene encoding FrpB, a major iron-regulated, outer membrane protein of *Neisseria gonorrhoeae*. *J Bacteriol* **177**, 2041-2049, doi:10.1128/jb.177.8.2041-2049.1995 (1995).

13 Schauer, K., Gouget, B., Carrière, M., Labigne, A. & de Reuse, H. Novel nickel transport mechanism across the bacterial outer membrane energized by the TonB/ExbB/ExbD machinery. *Mol Microbiol* **63**, 1054-1068, doi:10.1111/j.1365-2958.2006.05578.x (2007).

14 Grinter, R. *et al.* Structure of the bacterial plant-ferredoxin receptor FusA. *Nat Commun* **7**, 13308, doi:10.1038/ncomms13308 (2016).

15 Létoffé, S., Wecker, K., Delepierre, M., Delepelaire, P. & Wandersman, C. Activities of the *Serratia marcescens* heme receptor HasR and isolated plug and β-barrel domains: the β-barrel forms a heme-specific channel. *J Bacteriol* **187**, 4637-4645, doi:10.1128/JB.187.13.4637-4645.2005 (2005).

16 Elkins, C., Chen, C. J. & Thomas, C. E. Characterization of the *hgbA* locus encoding a hemoglobin receptor from *Haemophilus ducreyi*. *Infect Immun* **63**, 2194-2200 (1995).

17 Balhesteros, H. *et al.* TonB-dependent heme/hemoglobin utilization by *Caulobacter crescentus* HutA. *J Bacteriol* **199**, e00723-00716, doi:10.1128/JB.00723-16 (2017).

18 Funahashi, T. *et al.* An iron-regulated gene required for utilization of aerobactin as an exogenous siderophore in *Vibrio parahaemolyticus*. *Microbiology* **149**, 1217-1225, doi:10.1099/mic.0.26066-0 (2003).

19 Neugebauer, H. *et al.* ExbBD-dependent transport of maltodextrins through the novel MalA protein across the outer membrane of *Caulobacter crescentus*. *J Bacteriol* **187**, 8300-8311, doi:10.1128/JB.187.24.8300-8311.2005 (2005).

20 Eisenbeis, S., Lohmiller, S., Valdebenito, M., Leicht, S. & Braun, V. NagA-dependent uptake of *N*-acetyl-glucosamine and *N*-acetyl-chitin oligosaccharides across the outer membrane of *Caulobacter crescentus*. *J Bacteriol* **190**, 5230-5238, doi:10.1128/JB.00194-08 (2008).

21 Morris, J., Donnelly, D. F., O'Neill, E., McConnell, F. & O'Gara, F. Nucleotide sequence analysis and potential environmental distribution of a ferric pseudobactin receptor gene of *Pseudomonas* sp. strain M114. *Mol Gen Genet* **242**, 9-16 (1994).

22 Dean, C. R. & Poole, K. Cloning and characterization of the ferric enterobactin receptor gene (*pfeA*) of *Pseudomonas aeruginosa*. *J Bacteriol* **175**, 317-324, doi:10.1128/jb.175.2.317-324.1993 (1993).

23 Bitter, W., Marugg, J. D., de Weger, L. A., Tommassen, J. & Weisbeek, P. J. The ferric-pseudobactin receptor PupA of *Pseudomonas putida* WCS358: homology to TonB-dependent *Escherichia coli* receptors and specificity of the protein. *Mol Microbiol* **5**, 647-655, doi:10.1111/j.1365-2958.1991.tb00736.x (1991).

24 Koster, M., van de Vossenberg, J., Leong, J. & Weisbeek, P. J. Identification and characterization of the *pupB* gene encoding an inducible ferric-pseudobactin receptor of *Pseudomonas putida* WCS358. *Mol Microbiol* **8**, 591-601, doi:10.1111/j.1365-2958.1993.tb01603.x (1993).

25 Lynch, D. *et al.* Genetic organization of the region encoding regulation, biosynthesis, and transport

of rhizobactin 1021, a siderophore produced by *Sinorhizobium meliloti*. *J Bacteriol* **183**, 2576-2585, doi:10.1128/JB.183.8.2576-2585.2001 (2001).

26 Burkhard, K. A. & Wilks, A. Characterization of the outer membrane receptor ShuA from the heme uptake system of *Shigella dysenteriae*. Substrate specificity and identification of the heme protein ligands. *J Biol Chem* **282**, 15126-15136, doi:10.1074/jbc.M611121200 (2007).

27 Modrak, S. K., Melin, M. E. & Bowers, L. M. SucA-dependent uptake of sucrose across the outer membrane of *Caulobacter crescentus*. *J Microbiol* **56**, 648-655, doi:10.1007/s12275-018-8225-x (2018).

28 Blanvillain, S. *et al.* Plant carbohydrate scavenging through TonB-dependent receptors: a feature shared by phytopathogenic and aquatic bacteria. *PloS One* **2**, e224, doi:ARTN e22410.1371/journal.pone.0000224 (2007).

29 Noto, J. M. & Cornelissen, C. N. Identification of TbpA residues required for transferrin-iron utilization by *Neisseria gonorrhoeae*. *Infect Immun* **76**, 1960-1969, doi:10.1128/IAI.00020-08 (2008).

30 Thomas, C. E., Olsen, B. & Elkins, C. Cloning and characterization of *tdhA*, a locus encoding a TonB-dependent heme receptor from *Haemophilus ducreyi*. *Infect Immun* **66**, 4254-4262 (1998).

31 Calmettes, C. *et al.* The molecular mechanism of Zinc acquisition by the neisserial outer-membrane transporter ZnuD. *Nat Commun* **6**, 7996, doi:10.1038/ncomms8996 (2015).

32 Katayama Y, N. S., Nakamura M, Yano K, Yamasaki M, Morohoshi N, Haraguchi T. Cloning and expression of *Pseudomonas paucimobilis* SYK-6 genes involved in the degradation of vanillate and protocatechuate in *P. putida*. *Mokuzai Gakkaishi* **33**, 77-79 (1987).

33 Mori, K., Kamimura, N. & Masai, E. Identification of the protocatechuate transporter gene in *Sphingobium* sp. strain SYK-6 and effects of overexpression on production of a value-added metabolite. *Appl Microbiol Biotechnol* **102**, 4807-4816, doi:10.1007/s00253-018-8988-3 (2018).

34 Mori, K., Niinuma, K., Fujita, M., Kamimura, N. & Masai, E. DdvK, a novel major facilitator superfamily transporter essential for 5,5'-dehydrodivanillate uptake by *Sphingobium* sp. strain SYK-6. *Appl Environ Microbiol* **84**, doi:10.1128/AEM.01314-18 (2018).

35 Bolivar, F. & Backman, K. Plasmids of *Escherichia coli* as cloning vectors. *Methods Enzymol* **68**, 245-267 (1979).

36 Figurski, D. H. & Helinski, D. R. Replication of an origin-containing derivative of plasmid RK2 dependent on a plasmid function provided in *trans*. *Proc Natl Acad Sci U S A* **76**, 1648-1652, doi:10.1073/pnas.76.4.1648 (1979).

37 Blatny, J. M., Brautaset, T., Winther-Larsen, H. C., Karunakaran, P. & Valla, S. Improved broad-host-range RK2 vectors useful for high and low regulated gene expression levels in Gram-negative bacteria. *Plasmid* **38**, 35-51, doi:10.1006/plas.1997.1294 (1997).

38 Kaczmarczyk, A., Vorholt, J. A. & Francez-Charlot, A. Markerless gene deletion system for

Sphingomonads. *Appl Environ Microbiol* **78**, 3774-3777, doi:10.1128/AEM.07347-11 (2012).

39 Silva-Rocha, R. *et al.* The Standard European Vector Architecture (SEVA): a coherent platform for the analysis and deployment of complex prokaryotic phenotypes. *Nucleic Acids Res* **41**, D666-675, doi:10.1093/nar/gks1119 (2013).

40 Hayat, S., Peters, C., Shu, N., Tsirigos, K. D. & Elofsson, A. Inclusion of dyad-repeat pattern improves topology prediction of transmembrane β-barrel proteins. *Bioinformatics* **32**, 1571-1573, doi:10.1093/bioinformatics/btw025 (2016).

41 Almagro Armenteros, J. J. *et al.* SignalP 5.0 improves signal peptide predictions using deep neural networks. *Nat Biotechnol* **37**, 420-423, doi:10.1038/s41587-019-0036-z (2019).

42 Sievers, F. *et al.* Fast, scalable generation of high-quality protein multiple sequence alignments using Clustal Omega. *Molecular systems biology* **7**, 539, doi:10.1038/msb.2011.75 (2011).

43 Krogh, A., Larsson, B., von Heijne, G. & Sonnhammer, E. L. Predicting transmembrane protein topology with a hidden Markov model: application to complete genomes. *J Mol Biol* **305**, 567-580, doi:10.1006/jmbi.2000.4315 (2001).

44 Oeemig, J. S., Ollila, O. H. S. & Iwaï, H. NMR structure of the C-terminal domain of TonB protein from *Pseudomonas aeruginosa*. *PeerJ* **6**, e5412, doi:10.7717/peerj.5412 (2018).
